# Repurposing environmental DNA samples—detecting the western pearlshell (*Margaritifera falcata*) as a proof of concept

**DOI:** 10.1002/ece3.3898

**Published:** 2018-02-05

**Authors:** Joseph C. Dysthe, Torrey Rodgers, Thomas W. Franklin, Kellie J. Carim, Michael K. Young, Kevin S. McKelvey, Karen E. Mock, Michael K. Schwartz

**Affiliations:** ^1^ U.S. Forest Service National Genomics Center for Wildlife and Fish Conservation Rocky Mountain Research Station Missoula MT USA; ^2^ Ecology Center and Wildland Resources Department Utah State University Logan UT USA

**Keywords:** noninvasive sampling, quantitative PCR, sample archive

## Abstract

Information on the distribution of multiple species in a common landscape is fundamental to effective conservation and management. However, distribution data are expensive to obtain and often limited to high‐profile species in a system. A recently developed technique, environmental DNA (eDNA) sampling, has been shown to be more sensitive than traditional detection methods for many aquatic species. A second and perhaps underappreciated benefit of eDNA sampling is that a sample originally collected to determine the presence of one species can be re‐analyzed to detect additional taxa without additional field effort. We developed an eDNA assay for the western pearlshell mussel (*Margaritifera falcata*) and evaluated its effectiveness by analyzing previously collected eDNA samples that were annotated with information including sample location and deposited in a central repository. The eDNA samples were initially collected to determine habitat occupancy by nonbenthic fish species at sites that were in the vicinity of locations recently occupied by western pearlshell. These repurposed eDNA samples produced results congruent with historical western pearlshell surveys and permitted a more precise delineation of the extent of local populations. That a sampling protocol designed to detect fish was also successful for detecting a freshwater mussel suggests that rapidly accumulating collections of eDNA samples can be repurposed to enhance the efficiency and cost‐effectiveness of aquatic biodiversity monitoring.

## 1 INTRODUCTION

Environmental DNA (eDNA) sampling in aquatic environments has been lauded as a highly sensitive and efficient tool for assessing species presence, particularly for aquatic or semi‐aquatic species which are invasive (Dejean et al., 2012; Goldberg, Sepulveda, Ray, Baumgardt, & Waits, 2013), native but rare (McKelvey et al., 2016; Sigsgaard, Carl, Moller, & Thomsen, 2015; Wilcox et al., 2013), or cryptic and difficult to survey (Carim, Dysthe, Young, McKelvey, & Schwartz, 2017; Fukumoto, Ushimaru, & Minamoto, 2015). It has been applied to an array of taxa including frogs (Dejean et al., 2012; Ficetola, Miaud, Pompanon, & Taberlet, 2008; Goldberg, Pilliod, Arkle, & Waits, 2011), salamanders (Olson, Briggler, & Williams, 2012; Spear, Groves, Williams, & Waits, 2015), mollusks (Goldberg et al., 2013; Stoeckle, Kuehn, & Geist, 2015), crustaceans (Carim, McKelvey, Young, Wilcox, & Schwartz, 2016 2016), mammals (Padgett‐Stewart et al., 2015), lamprey (Carim et al., 2017), and bony fishes (Brandl et al., 2015; Mahon et al., 2013; Wilcox, Carim, McKelvey, Young, & Schwartz, 2015).

Although quantitative (qPCR)‐based eDNA sampling typically targets single species, each sample has the potential to provide multispecies occupancy data because a single sample potentially contains DNA of all animals present at or upstream from a location (Thomsen et al., 2012; Valentini et al., 2016). Given the dramatic and recent increase in eDNA surveys for single invasive or rare native species, eDNA sample collections are rapidly accumulating. Using eDNA to detect a single species typically only requires a portion of the total sample. Thus, if samples are properly preserved, archived, and annotated, these eDNA collections represent a trove of biodiversity data accessible at relatively low cost that can be repurposed for additional species.

One rapidly increasing collection of eDNA samples is associated with a range‐wide survey for bull trout (*Salvelinus confluentus*; Young et al., 2017). In this survey, environmental DNA samples were collected by dozens of collaborators throughout the Pacific Northwest and sent to the National Genomics Center for Wildlife and Fish Conservation, at the USDA Rocky Mountain Research Station, Missoula, MT. Here, the samples were extracted, analyzed for bull trout, archived, annotated, and stored in a central repository along with all metadata including sampling dates and locations. The bull trout detection results were then uploaded onto a publicly accessible database (https://www.fs.fed.us/rm/boise/AWAE/projects/BullTrout_eDNA/SurveyStatus.html) that has a user‐friendly interface allowing users to extract data associated with specific sampling locations. While this archive provides sample coverage across an extensive area, and therefore the potential to determine the occurrence of many species, the efficacy of repurposing eDNA samples is largely unknown, particularly when sampling strategies were initially designed to maximize detection rates for organisms with very different life histories.

Freshwater mussels are a diverse group of organisms with roughly 300 species native to North America and represent one of the most highly endangered and rapidly declining fauna on Earth (Haag, 2012). These extinctions and declines are often attributed to anthropogenic activities that impact water quality and foster the establishment of non‐native species (Bogan, 1993; Williams, Warren, Cummings, Harris, & Neves, 1993). In addition, freshwater mussels require native fish hosts for reproduction and dispersal, and so, freshwater mussel declines may also be tied to declining or changing fish communities. As declines continue, sensitive and reliable methods to assess distributions of freshwater mussel species are critical for focusing conservation efforts. Traditional mussel surveys are time‐intensive and require specialized expertise to provide reliable results, which discourages their application at broad scales. Thus, alternate approaches providing more rapid assessments with equal or greater sensitivity would be a significant contribution to conservation of freshwater mussels.

The western pearlshell mussel (*Margaritifera falcata*) is a freshwater bivalve native to western North America from California to southern Alaska and east to the headwaters of the Missouri River in Montana, the Snake River in Wyoming, and the Great Basin in Utah and Nevada (Nedeau, Smith, Stone, & Jepsen, 2009). Western pearlshell mussels are benthic organisms that are patchily distributed in low‐gradient habitats in clear, cold streams, with densities varying from locally abundant to very rare (Limm & Power, 2011; Stone, Barndt, & Gangloff, 2004). This species has been extirpated or is declining in many portions of its historical range (Nedeau et al., 2009), and intensive surveys to evaluate the distribution of this species have been recommended (Xerces Society, https://xerces.org/western-pearlshell/). Western pearlshell have been shown to emit significant quantities of organic matter likely to contain DNA in the form of feces (Limm & Power, 2011), and other possible sources of DNA may be released during molting, reproduction, mucus production, or decomposition posthumously (Deiner & Altermatt, 2014). However, their benthic habit, localized distribution, and low relative biomass could reduce detection probability with eDNA methods (Stoeckle et al., 2015).

Nonetheless, eDNA analysis has proven to be an invaluable tool for detecting a variety of taxa and has resulted in per site detection efficiencies exceeding traditional methods severalfold (Valentini et al., 2016; Wilcox et al., 2016). Reliable, whole‐basin eDNA sampling designs have been developed for other taxa (McKelvey et al., 2016) resulting in an accumulation of archived eDNA. While these samples were collected targeting the DNA of fishes, they likely captured DNA shed by other organisms, including western pearlshell, and could provide all or a significant portion of a western pearlshell survey minimizing the need for additional field effort.

Our primary goal was to design and validate an eDNA assay specific to the western pearlshell. Our secondary goal was to determine whether archived eDNA samples collected for detection of species with very different life histories (in this case, fish) could be repurposed to confirm the presence of western pearlshell at locations of historical occurrence. Ultimately, we demonstrate the utility of maintaining a well‐annotated archive of eDNA samples accessible from a central repository.

## 2 METHODS

We designed an environmental DNA assay specific to western pearlshell in three phases: *in silico*,* in vitro*, and *in vivo*. First, we compiled sequences of the cytochrome oxidase subunit I (COI) mitochondrial gene of this species (Mock, Brim Box, Chong, Furnish, & Howard, 2013; Table 1) and 10 other mollusk species (Table 1). We screened the sequences *in silico* using the *DECIPHER* package (Wright et al., 2014) in *R* v. 3.2.3 (R Core Development Team (2015)) and obtained candidate primers unique to western pearlshell. We aligned the primers with sequence data in MEGA 6.0 (Tamura, Peterson, Peterson, Filipski, & Kumar, 2013) and adjusted primer lengths and position in Primer Express 3.0.1 (Life Technologies) to optimize annealing temperatures (Table 2). In addition, we compared the primers to additional western pearlshell sequence data (GenBank accessions AY579126–579128 and DQ272374–272383) and identified a single nucleotide polymorphism 12 nucleotides from the 3′ end of the forward primer in four of the published sequences (accessions AY579126, AY579128, DQ272376, and DQ272382). To promote efficient amplification of all western pearlshell specimens, we incorporated a degenerate base (Kwok, Chang, Sninsky, & Wang, 1994; Wilcox et al., 2015) at this position in the forward primer. The resulting primers amplify a 106‐nucleotide fragment of the COI gene. Within this fragment, we visually identified an area unique to western pearlshell and designed a FAM‐labeled, minor‐groove‐binding, nonfluorescent quencher (MGB‐NFQ) probe (Table 2) to maximize nucleotide differences with nontarget sequences. We assessed the annealing temperature of the probe in Primer Express 3.0.1 (Life Technologies; Table 2) and examined potential secondary structure formation of the primer‐probe set using IDT OligoAnalyzer (https://www.idtdna.com/calc/analyzer). To confirm the specificity of the western pearlshell assay *in silico*, we performed BLAST searches on each primer and the probe.

**Table 1:** Species, sample size (*n*), and GenBank accession number for DNA sequences used for *in silico* eDNA marker development. Also included is the minimum number of base pair differences between each component of the eDNA marker and the nontarget sequences

Common name	Species name	*n*	GenBank accession	Forward primer mismatches	Reverse primer mismatches	Probe mismatches
Western pearlshell mussel	*Margaritifera falcata*	20	KF701440.1‐KF701459.1	0	0	0
Asian clam	*Corbicula fluminea*	4	AY943243.1; KC429132.1; KJ909515.1; U47647.1	4	13	8
California floater	*Anodonta californiensis* a	4	KF672876.1; KF672878.1‐KF672879.1; KF672892.1	6	6	6
Creeping ancylid	*Ferrissia rivularis*	2	KF737913.1‐KF737914.1	6	12	8
Fatmucket	*Lampsilis siliquoidea*	4	KC408784.1; KC408789.1; KC408793.1; KC408795.1	5	12	7
Freshwater pearl mussel	*Margaritifera margaritifera*	5	AY579129.1‐AY579130.1; DQ060171.1; JN243891.1; KC429108.1	3	3	4
Oregon floater	*Anodonta oregonensis* a	4	AY493503.1‐AY493504.1; DQ272359.1‐DQ272360.1	4	6	6
Western floater	*Anodonta kennerlyi* a	1	EU327351.1	4	7	6
Western ridged mussel	*Gonidea angulata*	4	AF231755.1; DQ272371.1‐DQ272372.1; KP795030.1	3	5	4
Winged floater	*Anodonta nuttalliana* a	4	DQ272365.1; DQ272368.1‐DQ272369.1; EU327355.1	7	5	5
Zebra mussel	*Dresseina polymorpha*	4	AM748984.1; EF414493.1; HM210079.1; U47653.1	8	18	7

a *Anodonta californiensis* and *Anodonta nuttalliana* are proposed to be a single species representing one clade of *Anodonta*, as is the case with *Anodonta oregonensis* and *Anodonta kennerlyi* (Chong et al., 2008; Mock et al., 2010). We keep them separate in this table to align with GenBank taxa designations and accessions.

**Table 2:** Environmental DNA assay for detecting western pearlshell mussel using qPCR

Assay component	Sequence (5′‐3′)	Tm (°C)	Optimal concentration (nM)
Forward primer	GGGTTTTGGTAATTGRCTTATTCCACT	59.8‐63.1	600
Reverse primer	ACAAGAAAAGAGCAGGCACAAGC	60.9	900
Probe	CCTTAACAATTTGAGGTTTTGATT	70	250

We also evaluated *in silico* the potential for cross‐amplification of common fish associates of the western pearlshell to confirm that these species did not pose the risk of primer competition, potentially limiting the efficacy of the assay. Thus, we compared the primers to genetic sequence data of fish species suggested in Nedeau et al. (2009) to host glochidia (parasitic larvae) of western pearlshell, including brook trout (*Salvelinus fontinalis*; accessions HQ961027–961028), brown trout (*Salmo trutta*; accessions HQ961021–961022), Chinook salmon (*Oncorhynchus tshawytscha*; accessions KU756212–756213), coho salmon (*O. kisutch*; accessions FJ164928–164929), cutthroat trout (*Oncorhynchus clarkii*; accessions JN027854–027855), rainbow trout (*Oncorhynchus mykiss*; accessions HQ961048–961049.1), and sockeye salmon (*O. nerka*; accessions HQ712704–712705).

To test the specificity of the assay *in vitro*, we performed qPCR analysis on DNA extracted from tissue of 23 western pearlshell specimens from 12 locations, as well as 23 nontarget mussel and fish species with which they co‐occur (Table 3). Western pearlshell tissue specimens were opportunistically collected during field sampling and were immediately preserved in 95% ethanol upon collection. Tissues of the nontarget mussel species were obtained from archived samples at Utah State University, Logan, UT. DNA was extracted using the DNeasy Tissue and Blood Kit (Qiagen, Inc) following the manufacturer's protocol. For the nontarget fish species, we used DNA archived at the National Genomics Center for Wildlife and Fish Conservation in Missoula, MT. Because the samples used in this study were from invertebrate organisms or from archived samples collected during previous studies, approval by an animal ethics committee was not required.

**Table 3:** Species used for *in vitro* testing of the western pearlshell eDNA assay. Origin refers to the waterbody for western pearlshell and to the state for all other samples

Common name	Species name	Sample size	Origin
Western pearlshell	*Margaritifera falcata*	1	Cat Spur Creek, ID
		2	East Fork Emerald Creek, ID
		6	North Fork Coeur d'Alene River, ID
		2	St. Joe River, ID
		2	St. Maries River, ID
		1	Clam Creek Slough, MT
		3	Clearwater River, MT
		1	East Fisher Creek, MT
		1	Five Mile Creek, MT
		2	Selway Creek, MT
		1	West Fork Rock Creek, MT
		1	Deschutes River, OR
California floater	*Anodonta californiensis*	2	OR, UT
Oregon floater	*Anodonta oregonensis*	2	OR
Western ridged mussel	*Gonidea angulata*	3	CA, OR, WA
Yukon floater	*Anodonta beringiana*	2	AK
Apache trout	*Oncorhynchus apache*	1	NM
Arctic grayling	*Thymallus arcticus*	2	MT
Atlantic salmon	*Salmo salar*	1	Commercial
Bonneville cutthroat trout	*Oncorhynchus clarki utah*	1	MT
Brook trout	*Salvelinus fontinalis*	1	VA
Brown trout	*Salmo trutta*	2	OR
Bull trout	*Salvelinus confluentus*	1	OR
Chinook salmon	*Oncorhynchus tshawytscha*	1	ID
Coastal cutthroat trout	*Oncorhynchus clarkii clarkii*	1	OR
Dolly Varden	*Salvelinus malma*	1	AK
Gila trout	*Oncorhynchus gilae*	1	NM
Brook lamprey	*Lampetra* spp.	1	OR
Muskellunge	*Esox masquinongy*	2	MN
Northern pike	*Esox lucius*	1	AK
Rainbow trout	*Oncorhynchus mykiss*	1	MT
Redband trout	*Oncorhynchus mykiss gairdneri*	1	OR
Westslope cutthroat trout	*Oncorhynchus clarki lewisi*	1	MT
Yellow perch	*Perca flavescens*	1	WA
Yellowstone cutthroat trout	*Oncorhynchus clarkii bouvieri*	1	WY

Each of these tissue‐derived DNA samples was analyzed with the western pearlshell assay on a StepOne Plus Real‐time PCR Instrument (Life Technologies) in 15‐μl reactions containing 7.5 μl 2× Environmental Master Mix 2.0 (Life Technologies), 900 nM of each primer, 250 nM probe, 4 μl DNA template (~0.4 ng), and 2.75 μl deionized water. The thermocycler profile included initial denaturation at 95°C for 10 min followed by 45 cycles of 95°C for 15 s and 60°C for 1 min. Experiments were set up inside a hood where qPCR consumables and pipettes were irradiated with UV for 1 h before setup. Each test included a no‐template control with distilled water substituted in place of DNA template to test for contamination.

We optimized primer concentrations (Table 2) in triplicate, 15‐μl reactions using the qPCR recipe above and varied concentrations of each primer (100, 300, 600, or 900 nM) for a total 16 unique combinations (Wilcox et al., 2015). Concentrations resulting in the earliest C_t_ value and a high endpoint fluorescence relative to the most concentrated level tested were selected for all subsequent analyses (Table 2). Using these optimized primer concentrations, we then performed a standard curve analysis to examine the sensitivity of the assay. The qPCR product was purified using GeneJET PCR Purification Kit (ThermoFisher Scientific) and quantified on a Qubit 2.0 Fluorometer (ThermoFisher Scientific). A seven‐level serial dilution (31 250, 6 250, 1 250, 250, 50, 10, and 2 copies per 4 μl) was created in sterile TE, and each level was analyzed in six replicates.

To validate the western pearlshell assay *in vivo*, we compiled western pearlshell occurrence data from the Middle and North Forks of the John Day River in Oregon (Brim Box et al., 2003, 2006) and from 16 streams in Montana and one in eastern Idaho (historical surveys; Stagliano, 2010, 2015). Historical surveys were conducted using traditional techniques such as snorkeling, SCUBA, aquascopes, and direct observation in Oregon in 2003 (Brim Box et al., 2003), and Montana and Idaho between 2007 and 2014 (Stagliano, 2010, 2015). We mapped these historical surveys onto our archive of eDNA sampling surveys to look for adjacency or overlap among survey types. We found eDNA surveys conducted in 2015 and 2016 targeting bull trout, smallmouth bass *Micropterus dolomieu*, and Arctic grayling *Thymallus arcticus* were near historical western pearlshell surveys (Table 4). Where sites from both surveys were overlapping or adjacent, we selected eDNA samples to re‐analyze with the western pearlshell assay and assigned each sample an expectation of DNA presence. All of the eDNA samples from the Middle and North Forks of the John Day River were expected to be positive for western pearlshell DNA in accordance with abundant detection in historical surveys (Brim Box et al., 2003) and more recent incidental observations of high mussel densities (Erika Rubenson, University of Washington, personal communication). Additionally, eDNA samples from Musselshell Creek in Idaho and Trail Creek in Montana were expected positive based on historical surveys (Stagliano, 2010). Samples from nine streams in Montana were expected to be negative for western pearlshell DNA based on historical surveys (Stagliano, 2010; Table 4).

**Table 4:** Collection information for *in vivo* testing of the western pearlshell assay. All samples were collected during surveys for other taxa (see text). Expectation of western pearlshell presence was based on proximity to historical locations (Brim Box et al., 2003, 2006; Stagliano, 2010, 2015). In the West Fork Rock Creek, all eDNA samples collected within the basin were analyzed for western pearlshell mussels to provide a formal comparison with basin‐wide historical mussel surveys (Stagliano, 2010, 2015; Figure 1). Site ID for the West Fork Rock Creek samples corresponds to sampling locations shown in Figure 1

Site ID	Waterbody (State)	Latitude	Longitude	Collection date	DNA detected
Reaches where western pearlshell mussels were present based on previous surveys					
MU‐01	Musselshell Creek, ID	46.369805	−115.7409	9/23/2015	Y
MF‐01	Middle Fork John Day River, OR	44.825133	−119.0109	7/21/2016	Y
MF‐02		44.805435	−118.9751	7/21/2016	Y
MF‐03		44.794485	−118.9528	7/21/2016	Y
MF‐04		44.786012	−118.9040	7/21/2016	Y
MF‐05		44.761035	−118.8602	7/21/2016	Y
MF‐06		44.717364	−118.8221	7/21/2016	Y
MF‐07		44.668549	−118.7115	7/21/2016	Y
MF‐08		44.641563	−118.6387	7/21/2016	Y
NF‐01	North Fork John Day River, OR	44.990971	−119.1040	7/20/2016	Y
NF‐02		45.008200	−119.0621	7/20/2016	Y
NF‐03		45.010071	−118.9964	7/20/2016	Y
NF‐04		44.997558	−118.9444	7/20/2016	Y
NF‐05		45.015043	−118.8728	7/20/2016	Y
NF‐06		44.986397	−118.7867	7/20/2016	Y
NF‐07		44.979284	−118.7285	7/20/2016	Y
TC‐01	Trail Creek, MT	45.656469	−113.7164	6/23/2015	Y
Reaches where western pearlshell mussels were absent based on previous surveys					
FL‐01	Flint Creek, MT	46.33762	−113.3205	7/18/2016	N
GR‐01	Grizzly Creek, MT	46.57349	−113.6577	8/14/2016	N
LB‐01	Little Blackfoot River, MT	46.42123	−112.4873	8/19/2015	N
LC‐01	Lost Creek, MT	46.20198	−112.9886	9/29/2015	N
MO‐01	Mormon Creek, MT	46.71898	−114.1407	9/21/2016	N
RA‐01	Ranch Creek, MT	46.52352	−113.6234	8/26/2016	N
RS‐01	Rattlesnake Creek, MT	46.94572	−113.9452	4/7/2015	N
ST‐01	Stony Creek, MT	46.33864	−113.6272	6/22/2016	N
WS‐01	Warm Springs Creek, MT	46.13576	−112.9626	9/29/2015	N
Results from the basin‐wide eDNA survey in West Fork Rock Creek					
BO‐01	Bowles Creek, MT	46.19227	−113.7491	7/20/2016	N
BO‐02		46.19341	−113.7533	7/20/2016	N
SB‐01	Sand Basin Creek, MT	46.19751	−113.7027	7/25/2016	Y
SB‐02		46.19344	−113.6943	7/25/2016	Y
SB‐03		46.18925	−113.6889	7/25/2016	Y
SB‐04		46.18138	−113.6884	7/25/2016	N
SB‐05		46.17457	−113.6881	7/25/2016	N
SB‐06		46.17130	−113.6771	7/25/2016	N
SB‐07		46.16728	−113.6752	7/25/2016	N
SB‐08		46.15910	−113.6789	7/25/2016	N
SB‐09		46.15189	−113.6888	7/25/2016	N
UN1‐01	Unnamed Tributary (1) to West Fork Rock Creek, MT	46.19227	−113.7163	7/21/2016	N
UN2‐01	Unnamed Tributary (2) to West Fork Rock Creek, MT	46.19228	−113.7052	7/26/2016	Y
UN3‐01	Unnamed Tributary (3) to West Fork Rock Creek, MT	46.19002	−113.6877	7/25/2016	N
UN3‐02		46.18613	−113.6796	7/25/2016	N
WF‐01	West Fork Rock Creek, MT	46.19658	−113.7039	7/25/2016	Y
WF‐02		46.19316	−113.7073	7/25/2016	Y
WF‐03		46.19369	−113.7175	7/21/2016	N
WF‐04		46.19861	−113.7222	7/21/2016	Y
WF‐05		46.19604	−113.7428	7/20/2016	N
WF‐06		46.19114	−113.7492	7/20/2016	N

**Figure 1:**
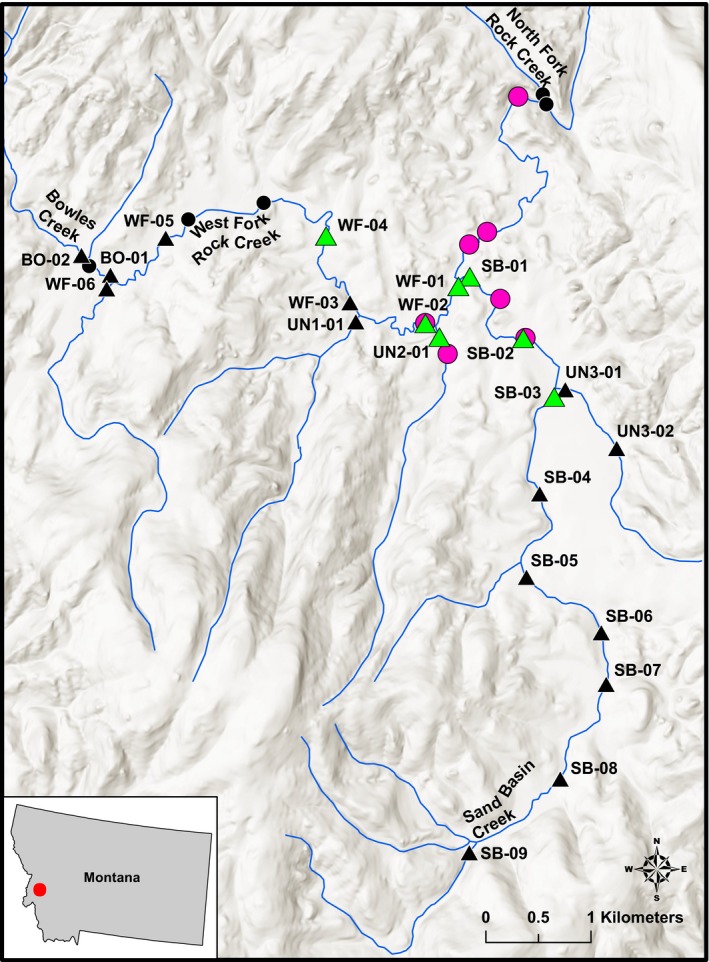
A map of the West Fork Rock Creek Basin, where both formal western pearlshell mussel surveys (Disks; Stagliano, 2010, 2015) and bull trout eDNA surveys (Triangles; Young et al., 2017) were conducted. Black symbols represent sites where surveys failed to detect western pearlshell mussels, magenta disks represent positive results in the western pearlshell mussel surveys, and green triangles represent positive results for western pearlshell mussels obtained by repurposing the collected eDNA samples. Repurposed eDNA samples labeled in this figure are shown in Table 4

Overlap among survey types was also found in the West Fork Rock Creek, Montana (Figure 1). Here, extensive surveys based on traditional techniques for western pearlshell (2014) and on eDNA techniques for bull trout (2016) were independently conducted, and precise location data were available for both surveys (Stagliano, 2015; Young et al., 2017). In this area, we directly compared the results of these basin‐level surveys.

All eDNA samples were collected following methods outlined in Carim, Dysthe, Young, McKelvey, and Schwartz (2016). Briefly, 5 l of water was pumped through a glass microfiber filter (pore size 1.5 μm) using a peristaltic pump, and the filter was stored in silica desiccant until extraction. DNA was extracted from each filter using the DNeasy Tissue and Blood Kit (Qiagen, Inc) following a modified protocol (Carim, Dysthe, et al., 2016). All eDNA was extracted in a room dedicated solely to this practice, and extracts were stored at −20°C until analyzed. Each sample was analyzed in triplicate 15‐μl reactions containing 7.5 μl Environmental Master Mix 2.0 (Life Technologies), optimized primer concentrations (Table 2), 250 nM probe, a TaqMan Exogenous Internal Positive Control (Life Technologies) including 1.5 μl of 10× IPC assay and 0.30 μl of 50× IPC DNA, and the remainder with deionized water. A no‐template control in which distilled water was substituted for DNA template was included in each analysis. For all qPCR experiments, a reaction was considered positive if the amplification curve crossed the assigned threshold during the exponential phase.

## 3 RESULTS

The results of the BLAST search indicated the potential for amplification of 10 nontarget mollusk species (Table 5). We aligned sequences of these species with the western pearlshell assay to examine the number of mismatches with each component of the assay. There were a minimum of eight mismatches, with at least one mismatch in each primer and four mismatches in the probe (Table 5). Comparisons of pearlshell DNA with DNA from fish species suggested in Nedeau et al. (2009) to host glochidia resulted in at least 18 mismatches in the primer region.

**Table 5:** Nontarget species resulting from BLAST searches of the forward and reverse primers

Common name	Species name	Range (cite)	*n*	GenBank accession	Forward primer mismatches	Reverse primer mismatches	Probe mismatches
Alabama pearlshell	*Margaritifera marrianae*	Alabama, eastern Gulf Coastal Plain, USA^a^	3	HM849096.1‐HM849098.1	2	1	5
Fine‐rayed pigtoe pearly mussel	*Fusconaia cuneolus*	Tennessee River, Cumberland Plateau, USA^a^	1	AY654998.1	3	3	5
Mapleleaf	*Quadrula quadrula*	Interior US, Nelson and Great Lakes basins, Gulf Coastal Plain, USA^a^	4	KX853979.1‐KX853982.1	1	2	7
Painted clubshell	*Pleurobema chattanoogaense*	Coosa basin, USA^b^	2	AY613829.1; AY655012.1	2	3	6
Ridged mapleleaf	*Quadrula rumphiana*	Mobile basin, eastern Gulf Coastal Plain, USA^a^	1	HM230409.1	1	2	7
Southern clubshell	*Pleurobema decisum*	Mobile basin; eastern Gulf Coastal Plain, USA^a^	4	AF232801.1; AY613832.1; AY655014.1; DQ383431.1	2	3	6
Southern mapleleaf	*Quadrula apiculata*	Gulf Coastal Plain, Tennessee River^a^	1	KT285648.1	1	2	8
Spengler's freshwater mussel	*Margaritifera auricularia*	Southwestern Europe^a^	4	AF303309.1; AF303311.1; AY579125.1; KC703969.1	3	1	5
N/A	*Dahurinaia dahurica*	Amur River system, Russia^c^	4	JX497736.1‐JX497739.1	2	4	4
N/A	*Solenaia carinata*	Yangtze River, China^a^	1	KX822669.1	2	2	4

a Distributions inferred from MUSSELp (2014).

b Distributions inferred from Campbell et al. (2008).

c Distributions inferred from Vinarski and Seddon (2011).

The assay successfully detected DNA in all samples extracted from western pearlshell tissue and did not detect DNA in any of the nontarget samples or no‐template controls. The standard curve amplified efficiently (100.60%, *r* ^2^ = 0.99, y‐intercept = 38.84, slope = −3.31) and had a limit of detection (defined as the lowest concentration with >95% amplification success; Bustin et al., 2009) at 10 copies per reaction, although DNA was detected in five of six replicates averaging two copies per reaction. The assay detected western pearlshell DNA at all sites proximal to historically identified populations (Table 4). The assay did not detect western pearlshell DNA in samples from nine streams in Montana where mussels were not previously observed in historical surveys and therefore not expected to occur (Table 4).

In the comparative surveys of the West Fork Rock Creek basin, the mussel locations based on eDNA and historical surveys were largely concordant in the area where both surveys were conducted (Figure 1). The eDNA survey detected western pearlshell DNA in five samples taken adjacent to sites where western pearlshell were historically observed (Figure 1, SB‐01, SB‐02, UN2‐01, WF‐01, WF‐02). Additionally, the eDNA survey detected western pearlshell DNA at one location ~2 km upstream from where western pearlshell have been previously detected during historical surveys (Figure 1, WF‐04) and at one location ~1 km upstream of the extent of the historical surveys (Figure 1, SB‐03). The eDNA survey did not detect DNA in four samples taken adjacent to sites where western pearlshell were historically absent (Figure 1, BO‐1, BO‐2, WF‐05, WF‐06). One eDNA sample that tested negative for the presence of western pearlshell DNA (Figure 1, WF‐03) was located about one km upstream and downstream of positive eDNA samples (Figure 1, WF‐02 and WF‐04, respectively). There were three sites where western pearlshell populations were documented in historical surveys that were beyond the downstream extent of the eDNA survey (Figure 1).

## 4 DISCUSSION

The assay we developed for the western pearlshell mussel is both efficient and specific, and effectively demonstrates the utility of repurposing eDNA sample collections. Environmental DNA samples collected for a previous independent eDNA survey from sites adjacent to traditionally identified populations produced consistent results (Table 4), even though none were specifically collected for western pearlshell. The samples were collected for a highly mobile, midwater species (bull trout), which has a very different life history than the sessile mussels. However, our results suggest current sampling, and analysis methods are sufficient to detect DNA of these very different taxa. In the eDNA samples where detections were anticipated based on historical occurrences, all produced positive detections; likewise, there were no detections where mussels were not anticipated based on historical absences.

The results from the comparative West Fork Rock Creek surveys show both the advantages and the disadvantages of repurposing eDNA samples for secondary species. In this case, the bull trout eDNA surveys were conducted uniformly throughout the upper basin at 1‐km intervals, with additional samples at the confluence of stream branches (McKelvey et al., 2016). Because the eDNA survey was uniform, we obtained positive western pearlshell results in a stream reach of the West Fork Rock Creek that did not immediately overlap with historical survey sites and in the Sand Basin Creek upstream from the extent of historical surveys. Further, the eDNA survey successfully identified the center of the mussel distribution adjacent to the confluence of the West Fork Rock Creek and Sand Basin Creek in agreement with historical surveys. However, the bull trout eDNA survey only sampled reaches identified as potential spawning and rearing habitat for bull trout (Isaak, Young, Nagel, Horan, & Groce, 2015). Thus, lower elevation mussel beds identified by historical surveys were not sampled during the bull trout eDNA surveys (Figure 1). This result is likely to be common: Existing eDNA samples collected to detect one species will most often not completely replace the need to collect new data at additional sites for another species. However, gleaning existing eDNA sample databases can be used as a first step to identify locations for additional survey efforts. As eDNA surveys for a wide variety of target species continue, we believe that future surveys for additional, secondary organisms can be accomplished with much‐reduced field effort through the repurposing of extant samples.

For broad‐scale repurposing to be effective, careful archiving of samples including precise information concerning both location and collection date is necessary. Further, samples must be carefully processed and stored to help prevent contamination and degradation. Laboratory benches and tools should be cleaned with bleach regularly, and all extractions should be carried out by skilled technicians in a room dedicated to eDNA extractions. Downstream experiments should be set up in a separate room preferably under a hood that can be irradiated with UV light to eliminate any residual DNA before and after setup. qPCR analyses should take place in a room separate from extraction and PCR setup to avoid the risk of contaminating samples with PCR product. To minimize degradation of eDNA samples prior to processing, we recommend storing sample filters in silica desiccant in a cool, dark location immediately upon collection, and processing or placing them a freezer within 2 weeks (Carim, McKelvey, et al., 2016). To minimize degradation after processing, one study in forensic science suggests that archived DNA extracts should be stored frozen in TE buffer at −80°C or below, or dried, amended with a trehalose additive, and stored at room temperature or −80°C (Smith & Morin, 2005). In addition, lo‐bind or siliconized storage tubes should be used to minimize DNA binding to the tube walls, and repeated freeze–thaw cycles should be avoided. While these are some general recommendations for long‐term storage of DNA, more research is needed to determine the temporal stability of DNA from environmental samples stored at these conditions.

We envision that in addition to augmenting current surveys, eDNA samples will ultimately provide a snapshot of historical conditions for retrospective surveys. However, to fully take advantage of the multitude of eDNA samples available for assaying nontarget species (e.g., repurposing), this will require carefully archiving metadata in an online database and archiving the sample itself in a way to avoid degradation and contamination. These needs are likely best met via dedicated institutions with proper curation experience and facilities. While these institutions require investment, the potential value of the archived data in terms of information and cost efficacy is enormous.

## CONFLICT OF INTEREST

None declared.

## AUTHOR CONTRIBUTIONS

TWF, KJC, KEM, KSM, MKY, and MKS conceptualized the study. JCD, TR, KJC, KEM, KSM, MKY, and MKS involved in methodology. JCD, TR, and TWF validated the study. JCD, TR, TWF, KSM, and MKY performed the formal analysis. JCD, TR, TWF, KJC, KEM, KSM, MKY, and MKS investigated the study. KEM, MKY, and MKS collected resources. JCD, TWF, KJC, KSM, MKY, and MKS drafted the manuscript. JCD, TR, TWF, KJC, KEM, KSM, MKY, and MKS revised the manuscript. TWS, KJC, KEM, KSM, MKY, and MKS involved in visualization. TWS, KEM, and MKS supervised the study. KEM, KSM, MKY, and MKS acquired funding.

## DATA ACCESSIBILITY

All data are fully available without restriction. Genetic sequence data were obtained from GenBank, and accession numbers are listed in the manuscript and tables.
